# Modelling of the transmission dynamics of carbapenem-resistant *Klebsiella pneumoniae* in hospitals and design of control strategies

**DOI:** 10.1038/s41598-022-07728-w

**Published:** 2022-03-09

**Authors:** Suttikiat Changruenngam, Charin Modchang, Dominique J. Bicout

**Affiliations:** 1 Biophysics Group, Department of Physics, Faculty of Science, Mahidol University, Bangkok, 10400 Thailand; 2 Institut Laue-Langevin, Theory group, 71 Avenue des Martyrs, 38042 Grenoble, France; 3 Centre of Excellence in Mathematics, CHE, Bangkok, 10400 Thailand; 4 Thailand Center of Excellence in Physics, CHE, 328 Si Ayutthaya Road, Bangkok, 10400 Thailand; 5 Univ. Grenoble Alpes, CNRS, UMR 5525, VetAgro Sup, Grenoble INP, TIMC, 38000 Grenoble, France

**Keywords:** Diseases, Infectious diseases

## Abstract

Carbapenem-resistant *Klebsiella pneumoniae* (CRKP) has emerged as a major threat to global public health. Epidemiological and infection controls associated with CRKP are challenging because of several potential elements involved in a complicated cycle of transmission. Here, we proposed a comprehensive mathematical model to investigate the transmission dynamics of CRKP, determine factors affecting the prevalence, and evaluate the impact of interventions on transmission. The model includes the essential compartments, which are uncolonized, asymptomatic colonized, symptomatic colonized, and relapsed patients. Additionally, symptomatic colonized and relapsed patients were further classified into subpopulations according to their number of treatment failures or relapses. We found that the admission of colonized patients and use of antibiotics significantly impacted the endemic transmission in health care units. Thus, we introduced the treatment efficacy, defined by combining the treatment duration and probability of successful treatment, to characterize and describe the effects of antibiotic treatment on transmission. We showed that a high antibiotic treatment efficacy results in a significantly reduced likelihood of patient readmission in the health care unit. Additionally, our findings demonstrate that CRKP transmission with different epidemiological characteristics must be controlled using distinct interventions.

## Introduction

*Klebsiella pneumoniae* (KP), a Gram-negative bacterium, is a member of the *Klebsiella* genus of *Enterobacteriaceae*. It is one of the most relevant opportunistic pathogens causing various nosocomial infections, such as bacteremia, pneumonia, wound infection, and intra-abdominal and urinary tract infection^1^. In health care settings, KP transmission can occur through direct person-to-person contacts such as contaminated hands of staff, contamination of the environment, or the use of contaminated medical equipment. Beta-lactams are the first-line treatment for KP infections. However, in recent years, KP has developed resistance to these antibiotics, including last-resort carbapenems. The overuse and/or misuse of such antibiotics has contributed to the emergence of carbapenem-resistant *Klebsiella pneumoniae* (CRKP)^2^. CRKP infections are associated with high morbidity and mortality^2–4^. The mortality of patients infected with CRKP, ranging from 30 to 44% and strikingly reaching 70% in the case of bacteremia, is three times higher than that of patients infected with susceptible KP strains^1,5–8^.

Carbapenem-resistant *Klebsiella pneumoniae* was originally reported in the United States during the late 1990s^9,10^. Since then, it has rapidly disseminated across countries and continents such as Canada, the UK, Spain, France, and India^4^. The incidence of CRKP has been increasing at an alarming rate in recent decades. The China Antimicrobial Resistance Surveillance Trial Program showed that the isolation rate of CRKP escalated from 0.9% in 2007 to 19.9% in 2018^11^. In 2019, the European Centre for Disease Prevention and Control reported that trends in a population-weighted mean percentage for resistance to carbapenems had significantly increased in European Union and European Economic Area countries over the last five years, with the three highest resistance percentages reported from Greece (58.3%), Romania (32.3%) and Italy (28.5%)^12^.

Controlling the spread of CRKP in health care units is challenging because the acquisition and transmission of CRKP is a convoluted process governed by several components. The admission of CRKP carriers is one of the most significant factors directly causing an increase in the prevalence of CRKP in hospitals. The carriage rate of CRKP on admission can vary from 3.4 to 38% depending on the settings^13–16^. Furthermore, most CRKP carriers are asymptomatic and can serve as the main reservoir of CRKP in hospitals^17–19^, causing ongoing spread in health care settings^20^. Additionally, several studies have shown that the percentage of asymptomatic carriers who show no signs or symptoms and do not progress into infections can vary over a considerable range, from 0.3 to 69.5%^16,21–23^. Therefore, without active surveillance of the CRKP prevalence, we cannot establish contact precautions among the carriers in a timely manner to contain CRKP transmission*.*

Treatment of infections associated with CRKP is evidently problematic with extremely high failure rates, resulting in an increase in the hospital length of stay^24–27^. Additionally, despite receiving appropriate antibiotic therapy, patients occasionally relapse with the same strain of CRKP^28–33^. The percentage of patients with subsequent relapsing infections can reach almost 17%^34–37^. Furthermore, among patients with more than one episode of bacteremia, 39.5% of them had a relapsed infection with the same strain^38^. Accordingly, rehospitalization of these relapsed patients is another integral part that seriously contributes to the accelerated transmission of this pathogen^24,39,40^. Mathematical models have been extensively employed to examine the spread of nosocomial pathogens and estimate the impact of intervention^41–48^. One of the basic and popular modelling frameworks is the compartmental model in which the population of interest is divided into separated compartments based on their infection status^47^. In the past few years, the models were extended to incorporate contact precautions^48^, environment^44,47^, or antibiotic use^43^ to gain insight into the spread of pathogens, such as methicillin-resistant *Staphylococcus aureus* (MRSA), *Acinetobacter baumannii* and vancomycin-resistant *enterococci* (VRE). However, very few studies have focused on CRKP transmission^49,50^*.* In those studies, patients were merely categorized into uncolonized and colonized patients. Only the isolation of colonized patients, hand hygiene compliance and contact precautions were interventions concerned in their models to assess the impact of measures to control the spread. Additionally, none of those studies considered the effect of antibiotic treatment on epidemics. To examine the sophisticated mechanism underlying CRKP transmission, the attributable components corresponding to the dissemination of CRKP must be incorporated into the model.

In this work, we constructed a comprehensive model to investigate the mechanisms by which these pathogens spread within health care settings and to evaluate the extent to which infection control measures contribute to CPKP confinement. Unlike previous models on KP, our analysis includes the essential components, which are uncolonized, asymptomatic colonized, symptomatic colonized, and relapsed patients. The symptomatic colonized and relapsed patients were further differentiated into different classes according to the number of times that a patient experienced treatment failure in the hospital.

We fundamentally examined the impact of admission of colonized patients on the endemic prevalence of CRKP and assessed the effect of antibiotic treatment on transmission. In this study, we defined the treatment efficacy, which considers the treatment duration and probability of successful treatment, both of which can affect the prevalence of CRKP in hospitals^43^. Additionally, we calculated the probability distribution of patients experiencing a different number of treatment failures or relapses in the hospital. Finally, this study represents the first attempt to obtain disease control guidelines under different treatment scenarios. This guideline should be beneficial for treatment decision designs that effectively prevent or reduce the spread of CRKP in hospitals.

## Results

### The CRKP model formulation

**Figure 1:**
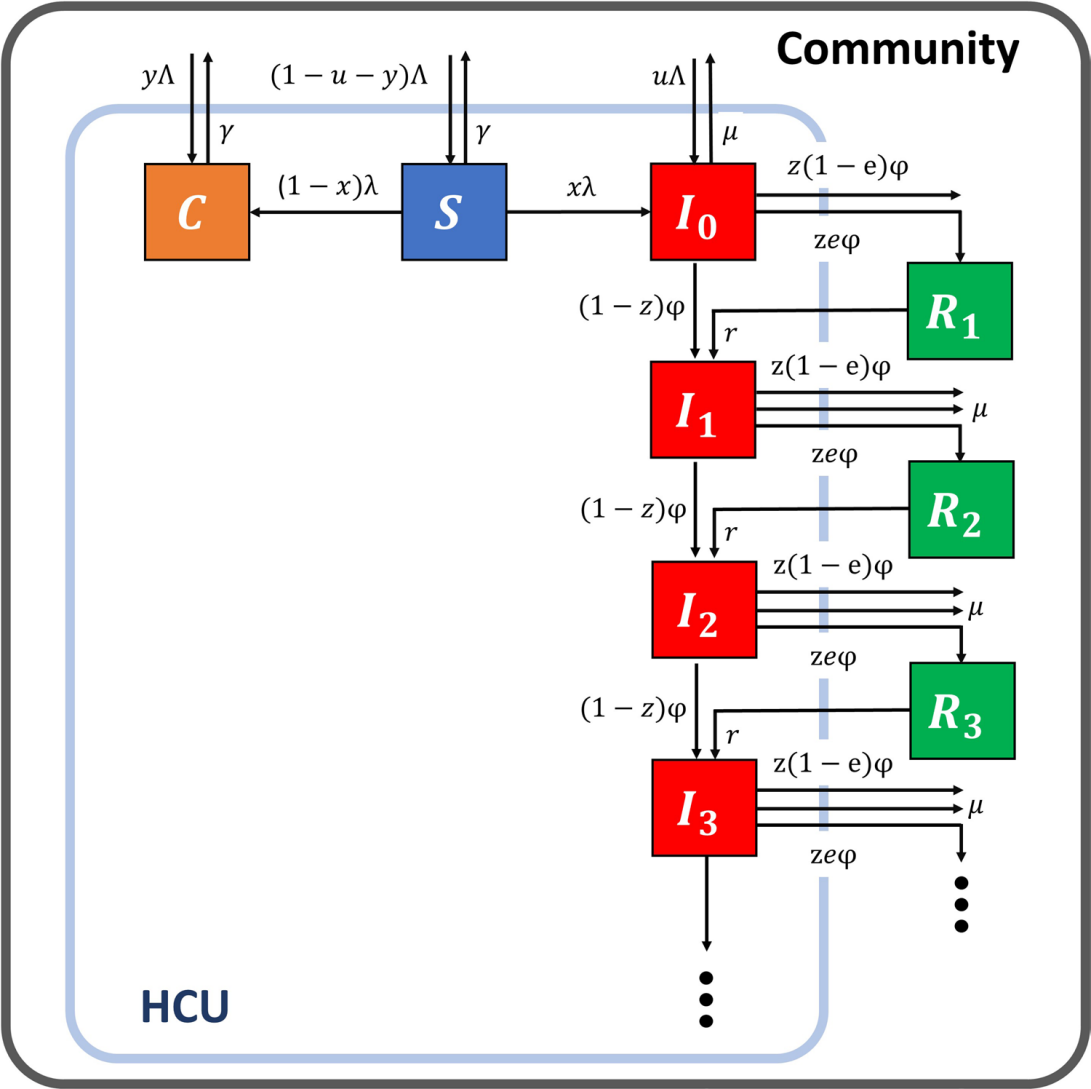
Schematic of the kinetic transmission model of carbapenem-resistant *Klebsiella pneumoniae* (CRKP) in a health care unit (HCU).

The transmission dynamics of carbapenem-resistant *Klebsiella pneumonia* (CRKP) within a health care unit (HCU) involves the transmission of the pathogen between two distinct groups of the population—namely, patients and staff. Patients are usually considered hosts, whereas staff act as vectors transmitting CRKP from patients to patients. In the proposed model (Fig. 1), patients are classified into four epidemiological classes based on their CRKP infection status: uncolonized ($$S$$), asymptomatic colonized ($$C$$), symptomatic colonized ($$I)$$, and relapsed $$\left( R \right)$$ patients. Because asymptomatic colonized patients cannot be identified without active surveillance, they are, therefore, treated as ordinary patients as if they were not colonized by CRKP. By contrast, symptomatic patients are easier to identify and will be treated under contact precautions such as the use of gloves, gowns, private rooms, or cohort rooms housing only symptomatic colonized patients with the same strain. Relapsed patients are those who had previously received successful treatment but were readmitted to the hospital because of a relapse of the infection. Here, successful treatment refers to a treatment in which a patient is cured and no longer exhibits any more clinical signs of symptoms after the treatment. Additionally, staff are divided into two classes: uncontaminated $$\left( {S_{S} } \right)$$ and contaminated $$\left( {C_{S} } \right)$$ who have not been (or are no longer) and have been contaminated with CRKP, respectively. For simplicity and because diseased staff are assumed to be self-isolated, the staff compartments are not explicitly shown in the kinetic scheme in Fig. 1.

In the kinetic transmission model of CRKP, uncolonized patients acquire CRKP following contacts with contaminated staff at a rate $\lambda$:

1 $$\begin{array}{*{20}l} {\lambda = ab\left( {1 - \delta } \right)\frac{{C_{s} }}{{N_{s} }},} \\ \end{array}$$

where $a$ is the daily number of contacts for a patient, $b$ is the probability that an uncolonized patient acquires CRKP after contact with contaminated staff, $\delta$ is the precaution compliance, and $N_{S} = S_{s} + C_{s}$. At the early stage of the acquisition of CRKP, they are all viewed as asymptomatic colonized patients. A fraction $x$ of them then become ‘short-term’ asymptomatic colonized patients who subsequently progress into the symptomatic colonized class after a certain time^51,52^. In the model, the time to develop infection is assumed to be very short; therefore, they immediately become symptomatic colonized patients ($I$). Although this assumption can affect the transmission dynamics by shortening the time course of transmission, it does not change the prevalence of colonized patients at equilibrium. The remaining fraction becomes (long-term) asymptomatic colonized ($C$) patients who never progress into infection during their hospital stay^13,16,17,19,23,52–55^. Symptomatic colonized patients are then treated with antibiotics at a rate $\varphi$ for which treatment either succeeds with probability z in curing the patients or fails with probability $1 - z$. Among cured patients discharged from the hospital, a fraction $e$ of them subsequently develop a relapse of infection and will be rehospitalized at a relapse rate $r$^56,57^. Symptomatic colonized, $I_{k}$, and relapsed patients, $R_{k}$, are distinguished and monitored by the history index *k* counting the number of times a patient has already experienced failed treatments or relapses. For example, $I_{0}$ and $I_{1}$ represent the number of symptomatic colonized patients who have received no and one antibiotic treatment, respectively. Natural decolonization of CRKP is excluded in the model because the duration of natural decolonization is much longer than the other time scales (e.g., length of stay in the hospital)^58–60^. In the absence of treatment, symptomatic colonized patients die from the infection at a rate $\mu$. The total admission rate of patients in the hospital is Λ, among which fractions $u$ and $y$ are symptomatic and asymptomatic colonized patients ($k = 0{ }$), respectively. The discharge of uncolonized patients and asymptomatic colonized patients occurs at the same rate $\gamma$ because they are indistinguishable.

Similarly, uncontaminated staff become contaminated with CRKP after contacting asymptomatic or symptomatic colonized patients at a rate $\lambda_{S}$:

2 $$\begin{array}{*{20}l} {\lambda_{s} = amb_{s} \left( {1 - \delta } \right)\frac{{\left( {C + pI} \right)}}{N},} \\ \end{array}$$

where $I = \mathop \sum \nolimits_{k = 0}^{{}} I_{k}$ is the total number of symptomatic colonized patients; $N$ is the total number of patients in the hospital,$N =$ $S + C + I$; $m$ is the patient density—i.e., the ratio of the total number of patients to that of staff in the hospital; and $b_{s}$ is the probability that an uncontaminated staff becomes contaminated after contact with colonized patients. However, staff engaged in the treatment or care of symptomatic colonized patients must follow contact precautions to prevent or reduce the transmission of pathogens. The probability of transmitting the pathogen from symptomatic colonized patients to staff is controlled by the effectiveness of contact precautions ($p$): $0 \le p \le 1$. For example, $p = 0$ and $p = 1$ indicate that contact precautions—e.g., using gloves—absolutely can and cannot protect transmitting pathogens between patients and staff, respectively. However, contaminated staff can be decontaminated at a rate $\left( {1 - \delta } \right)\alpha_{min} + \delta \alpha_{max}$, where $\alpha_{min}$ and $\alpha_{max}$ are the minimum and maximum decontamination rates, respectively.

To describe the dynamics of CPKP transmission, we exploited the structure of mathematical models for vector-borne diseases^46,61^. In this model, patients are considered target hosts, whereas staff are considered vectors transmitting CRKP from patients to patients. The change in the number of individuals in each subpopulation is calculated using ordinary differential equations. For patients, the dynamics of the transmission are described as follows:

3 $$\begin{array}{*{20}l} {\frac{dS}{{dt}} = \left( {1 - u - y} \right)\Lambda - \lambda S - \gamma S,} \\ \end{array}$$

4 $$\begin{array}{*{20}l} {\frac{dC}{{dt}} = y\Lambda + \left( {1 - x} \right)\lambda S - \gamma C,} \\ \end{array}$$

5 $$\begin{array}{*{20}l} {\frac{{dI_{0} }}{dt} = u\Lambda + x\lambda S - \left( {\varphi + \mu } \right)I_{0} ,} \\ \end{array}$$

6 $$\begin{array}{*{20}l} {\frac{{dI_{k} }}{dt} = \left( {1 - z} \right)\varphi I_{k - 1} + rR_{k} - \left( {\varphi + \mu } \right)I_{k} ,} \\ \end{array}$$

7 $$\begin{array}{*{20}l} {\frac{{dR_{k} }}{dt} = ze\varphi I_{k - 1} - rR_{k} ,} \\ \end{array}$$

For staff, the rates of changes of individuals in each compartment are described as follows:

8 $$\begin{array}{*{20}l} {\frac{{dS_{S} }}{dt} = - \lambda_{S} S_{s} + \left[ {\left( {1 - \delta } \right)\alpha_{min} + \delta \alpha_{max} } \right]C_{s} ,} \\ \end{array}$$

9 $$\begin{array}{*{20}l} {\frac{{dC_{S} }}{dt} = \lambda_{S} S_{s} - \left[ {\left( {1 - \delta } \right)\alpha_{min} + \delta \alpha_{max} } \right]C_{s} .} \\ \end{array}$$

The basic reproduction number ($R_{0}$) is an important epidemiologic metric used to describe the transmissibility of infectious disease. It provides the number of secondary cases generated by a colonized individual during his or her infectious period. $R_{0}$ can be computed as follows:

10 $$\begin{array}{*{20}l} {R_{0} = \left[ {\frac{{mbb_{s} a^{2} \left( {1 - \delta } \right)^{2} }}{{\left( {1 - \delta } \right)\alpha_{min} + \delta \alpha_{max} }}} \right]\left[ {\frac{1 - x}{\gamma } + \frac{px}{{\left( {1 - e} \right)z\varphi + \mu }}} \right].} \\ \end{array}$$

Details on the derivation of the basic reproduction number are described in the Methods section. The descriptions and values of all parameters used in the model are summarized in Table 1.

**Table 1:** Variables and parameters of the model.

Symbol	Definition	Value [range]	Unit	References
$u$	Fraction of symptomatic colonized patients at admission	0 [0–1]	–	Assumed
$y$	Fraction of colonized patient at admission	0 [0–1]	–	Assumed
$\gamma$	Discharge rate of patients	1/8.8 [1/18–1/4.4]	1/day	^19,50,51,53,62–66^
$a$	Total number of contacts that a patient acquires per day	8 [8–13.8]	– 1/day	^46,67^
$N$	Number of patients in a hospital	120	–	Assumed
$m$	Patient density (ratio of the no. patients to staff)	4 ^1–8^	–	^43,46,49,67–70^
$b$	Probability of a patient acquiring CRKP after contacting a contaminated staff	0.025 [0.01–0.42]	-–	^46,47,61,67^
$b_{S}$	Probability of an uncontaminated staff becoming contaminated after contacting a colonized or symptomatic colonized patient	0.11 [0.1–0.45]	–	^45–47,49,50,61,67^
$p$	Contact precaution effectiveness	0.63 [0–1]	–	Assumed
$\alpha_{min}$	Minimum decontamination rate	2	1/day	Assumed
$\alpha_{max}$	Maximum decontamination rate	24		
$\delta$	Precaution compliance	0–1	–	Assumed
$x$	Fraction of uncolonized patients becoming symptomatic colonized patients	0.2 [0.09–0.56]	–	^13,17,19,23,51–54,71–73^
$\lambda$	Force of infection of uncolonized patients becoming colonized after contacting contaminated staff	Follow Eq. (1)	–	–
$\lambda_{s}$	Force of infection of uncontaminated staff becoming contaminated after contacting colonized patients	Follow Eq. (2)	–	–
$z$	Probability of successful treatment	0–1	–	Assumed
$e$	Probability of a patient with a subsequent relapse of infection	Equation (19)	–	–
$e_{max}$	Maximum value of $e$	0.8	–	Assumed
$\varphi$	Treatment rate	1/14 [1/28–1/7]	1/day	^35–37,39,74–78^
$r$	Readmission rate due to a relapse of infection	1/16 [1/84–1/8.5]	1/day	^30,35,39,40,56,57,75,76,79^
$\mu$	Death rate associated with CRKP	0.0178 [0.014–0.073]	1/day	^5,6,8,80–84^

### Prevalence drivers

The admission of colonized patients from the community or other health care establishments direct affects the incidence and prevalence of CRKP carriage and infections within the considered hospital^33,85^. In this section, we run simulations of the model without antibiotic use to investigate the effects of exogenous patients. The degree of the admitted colonized patients is represented by the incoming prevalence ($P_{in}$), which is defined as the combination of a fraction of symptomatic colonized ($u$) and asymptomatic colonized patients ($y$) admitted to the hospital: $P_{in} = u + y$, such that $P_{in} \le 1$. For example, $P_{in} = 0$ and $P_{in} = 1$ represent no and only colonized patients being hospitalized, respectively. The effects or impacts on the epidemics within the hospital are represented by the prevalence ($P$) and the proportion of symptomatic to all colonized patients ($q$) as follows:

11 $$\begin{array}{*{20}l} {\left\{ {\begin{array}{*{20}l} {P = \frac{C + I}{N},} \\ {q = \frac{I}{C + I},} \\ \end{array} } \right. } \\ \end{array}$$

such that $qP$ provides the fraction of symptomatic colonization among all patients. Because the transmission is driven by both endogenous and exogeneous patients, $P$ and $q$ are functions of both $R_{0}$ and $P_{in}$ and can be expressed as follows:

12 $$\begin{array}{*{20}l} {\left\{ {\begin{array}{*{20}l} {P = \left( {1 - \theta_{1} } \right)P_{0} + \theta_{1} P_{1} ,} \\ {q = \left( {1 - \theta_{2} } \right)q_{0} + \theta_{2} q_{1} .} \\ \end{array} } \right.} \\ \end{array}$$

where $P_{0}$ is the (within hospital) prevalence in the case of zero incoming colonized patients $(P_{in} = 0$) and $P_{1}$ is the (within hospital) prevalence in the case when all admitted patients into the hospital are colonized $(P_{in} = 1$). Thus, $P_{1}$ is always one. The first term in the expression of $P$ represents the impact of prevalence generated by colonized patients only within the hospital, and the second term represents the impact of incoming prevalence. $1 - \theta_{1}$ and $\theta_{1}$ are weights accounting for the contribution of $P_{0}$ and $P_{1}$ to the prevalence $P$, respectively. Similarly, the proportion ($q$) can be described as the weighted combination of the proportion generated by within-hospital and incoming patients, where $q_{0}$ and $q_{1}$ are the proportions with $P_{in} = 0$ and $P_{in} = 1$, respectively. Likewise, $1 - \theta_{2}$ and $\theta_{2}$ are weights accounting for the contribution of $q_{0}$ and $q_{1}$ in $q$. The weights $\theta_{1}$ and $\theta_{2}$ can be obtained from simulations by inverting Eq. (12) as follows:

13 $$\begin{array}{*{20}l} {\left\{ {\begin{array}{*{20}l} {\theta_{1} = \frac{{P - P_{0} }}{{P_{1} - P_{0} }},} \\ {\theta_{2} = \frac{{q - q_{0} }}{{q_{1} - q_{0} }}.} \\ \end{array} } \right.} \\ \end{array}$$

**Figure 2:**
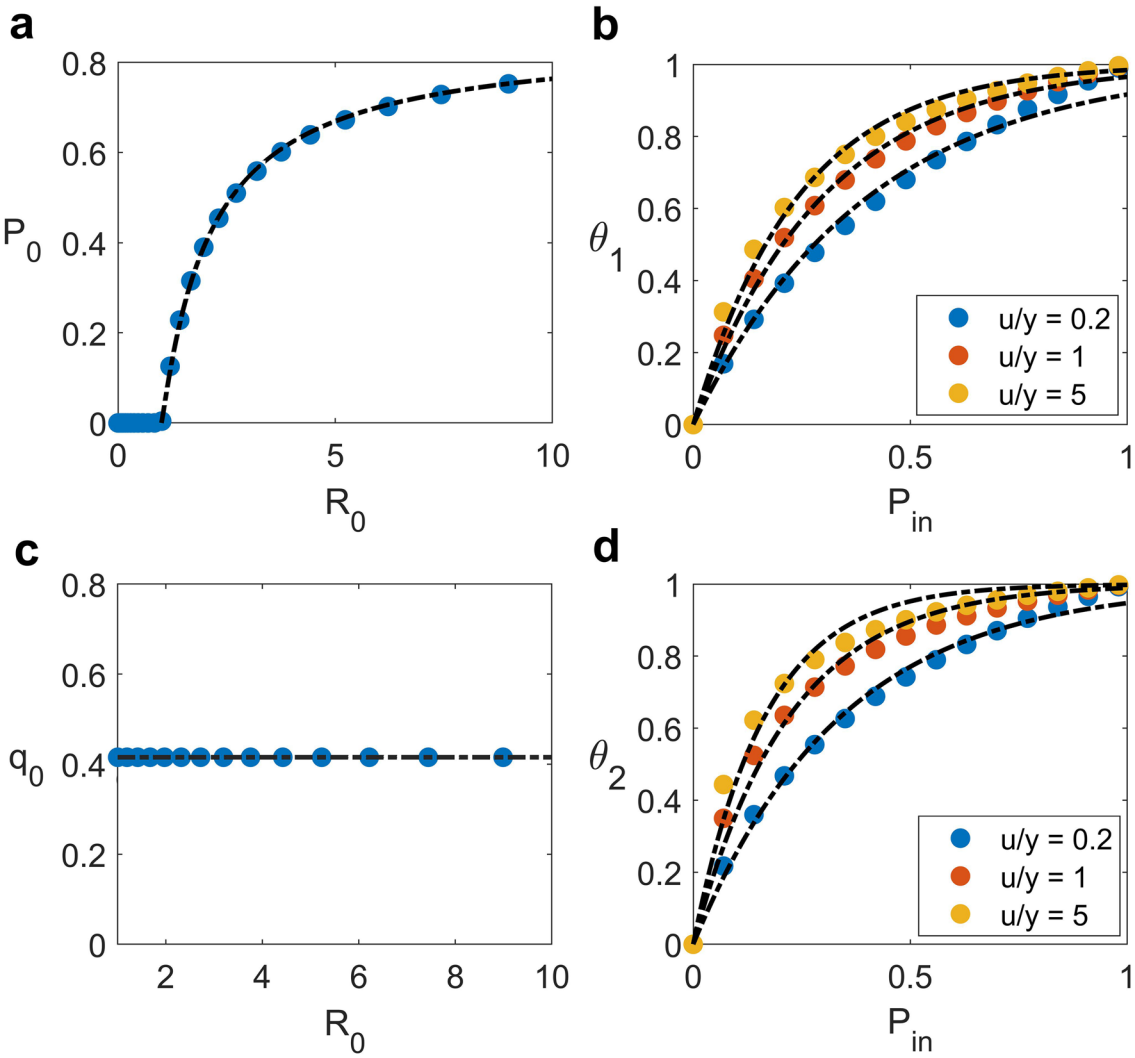
Prevalence and fraction of colonized patients. (**a**) Hospital prevalence and (**c**) fraction of colonized patients as a function of ${R}_{0}$ with zero incoming prevalence,${P}_{in}=0$**.** (**b**) Contribution weight for prevalence and (**d**) the fraction of colonized patients as a function of ${P}_{in}$. The disease is assumed to spread with ${R}_{0}=1.5$. Different colours represent different ratios of fractions of symptomatic colonized to asymptomatic colonized patients admitted to the hospital. Black-dash lines are the best fit for simulation data.

From Fig. 2a, the $P_{0}$ used to represent the intrinsic spread in the hospital is zero for $R_{0} < 1$, and it continuously increases for $R_{0} > 1$ with the expression of $P_{0}$ as a function of $R_{0}$ given in Table 2. Figure 2b shows that $\theta_{1}$ increases from zero at $P_{in} = 0$ to one at $P_{in} = 1$, and it is slightly higher for a larger ratio of symptomatic colonized patients admitted to the hospital (or higher $u/y$) (see Table 2 for the expression of $\theta_{1}$). In the absence of treatment, symptomatic colonized patients will stay longer in the hospital until they die at a rate $\mu$, while asymptomatic colonized patients will be discharged at a rate $\gamma$. Therefore, an increase in the admission of symptomatic colonized patients affects the prevalence more than that of asymptomatic colonized patients.

**Table 2:** Formulas and R-squares from curve fittings.

Fitting equations			R-square
$P_{0} = 0.8495\left( {1 - \frac{1}{{R_{0} }}} \right)^{1.095}$		for $u = y = 0$	0.9996
$q_{0} = \frac{1}{{1 + \left( {\frac{1 - x}{x}} \right)\left( {\frac{\mu }{\gamma }} \right)}} = 0.4154$		for $u = y = 0$	1.0000
$\theta_{1} =$	$1 - {\text{exp}}\left( { - 2.484P_{in} } \right)$	for $u/y = 0.2$	0.9866
	$1 - {\text{exp}}\left( { - 3.365P_{in} } \right)$	for $u/y = 1.0$	0.9940
	$1 - {\text{exp}}\left( { - 4.157P_{in} } \right)$	for $u/y = 5.0$	0.9870
$\theta_{2} =$	$1 - {\text{exp}}\left( { - 2.943P_{in} } \right)$	for $u/y = 0.2$	0.9927
	$1 - {\text{exp}}\left( { - 4.538P_{in} } \right)$	for $u/y = 1.0$	0.9776
	$1 - {\text{exp}}\left( { - 6.080P_{in} } \right)$	for $u/y = 5.0$	0.9559

Regarding the effect of admission on the proportion of symptomatic to all colonized patients, ${q}_{0}$ is constant and independent of ${R}_{0}$, as expected (Fig. 2c, see Table 2 for the analytical expression of ${q}_{0}$). The behaviour of ${\theta }_{2}$ as a function of ${P}_{in}$ and $u/y$ is similar to that of ${\theta }_{1}$ (see Fig. 2d and Table 2). This reason is that an increase in the proportion of symptomatic to all colonized patients is significantly affected by the fraction of symptomatic colonized patients admitted to the hospital. Note that the relationships among those parameters were investigated by fitting the simulation data with mathematical expressions. For each graph, the formulas and r-squares obtained from the best fit to simulation data are all summarized in Table 2.

### Treatment efficacy

To estimate the impact of treatment on the progression and prevalence of infection, how the effectiveness of this treatment is assessed must first be clarified. The length of hospital stay is one of the risk factors facilitating the spread of CRKP in hospitals. Patients with prolonged hospital duration are more likely to transmit the pathogens to uncolonized patients and vice versa. Additionally, the probability of successful treatment is another undeniable factor controlling the number of symptomatic colonized patients in the hospital. The lower is the successful treatment probability, the more unfavourable are the outcomes—e.g., a relapse of infection and treatment failure. Patients with those adverse outcomes can subsequently become reservoirs of CRKP in the hospital setting. In this study, the treatment was applied only to symptomatic colonized patients. To investigate the effects of the treatment on transmission, we introduced the treatment efficacy ($TE$) indicator defined as the ratio of the rate of successful treatment without relapse to the total removal rate of patients, including death mortality, as follows:

14 $$\begin{array}{*{20}l} {TE = \frac{{\left( {1 - e} \right)z\varphi }}{{\left( {1 - e} \right)z\varphi + \mu }} = \frac{{\left( {1 - e} \right)zf}}{{\left( {1 - e} \right)zf + \left( {1 - f} \right)}}.} \\ \end{array}$$

The indicator $TE = TE\left( {z,f|,e_{m} ,\nu } \right)$ is a two-dimensional function of $z$ (indicating the effectiveness in curing patients) and $f$ (measuring the probability of leaving the hospital alive), defined as the fraction of symptomatic colonized patients who escape death because of treatment,

15 $$\begin{array}{*{20}l} {f = \frac{\varphi }{\varphi + \mu }.} \\ \end{array}$$

Because the probabilities $z$ and $e$ are not independent, we assume that the relationships between $z$ and $e$ can be described by the relation:

16 $$\begin{array}{*{20}l} {e = e_{m} \left( {1 - z} \right)^{\nu } ,} \\ \end{array}$$

where $e_{m}$ is the maximum value of $e$ and $\nu$ is the shape parameter. Equation (16) can be regarded as the characteristic patient response to an antibiotic. When $z$ is high, $e$ is small and vice versa. Thus, most patients are likely to be cured and discharged from hospitals, and few of them relapse.

**Figure 3:**
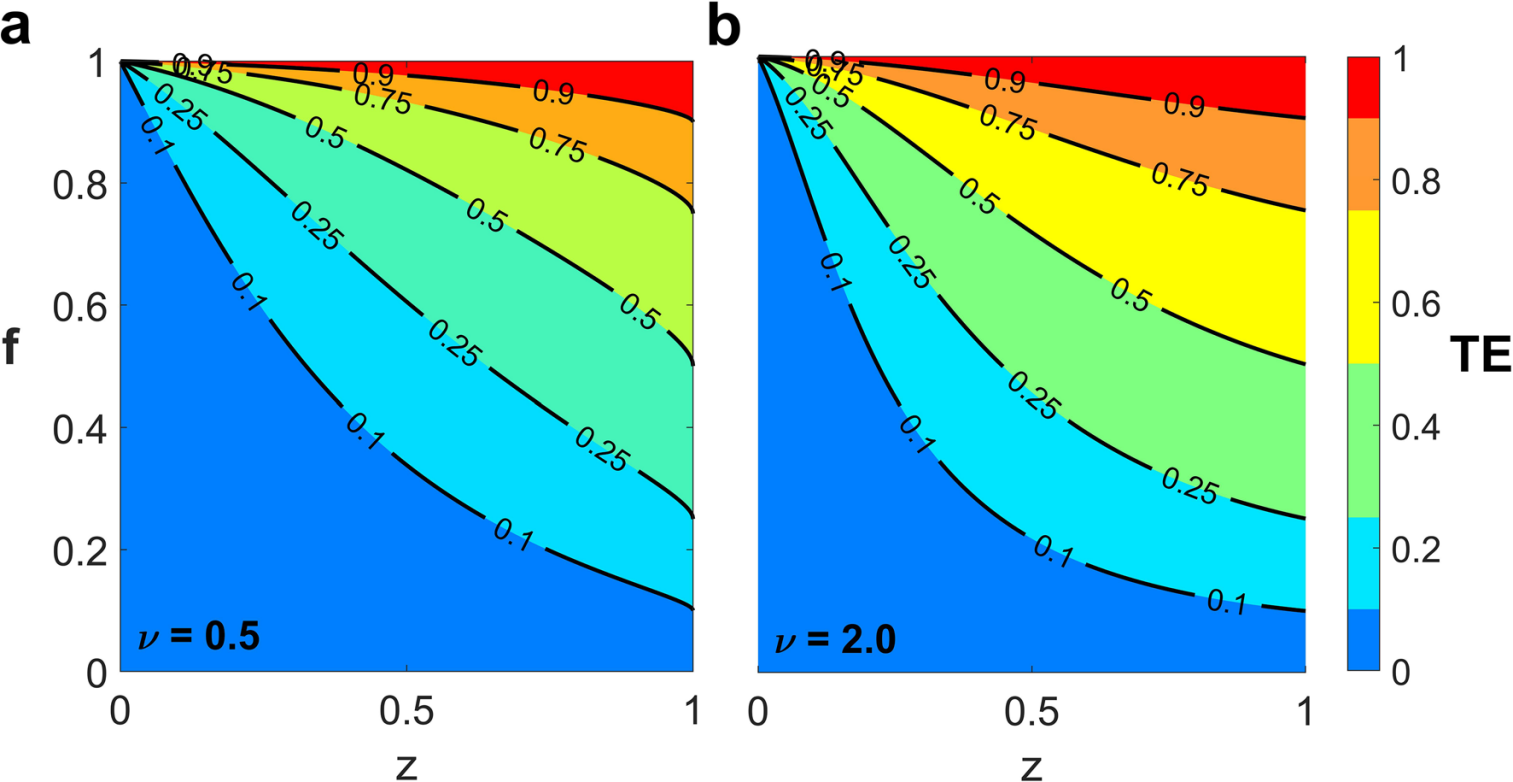
Contours of treatment efficacy. The treatment efficacy ($TE$) is plotted in (z, f) space with $\nu$ = 0.5 (**a**) and 2.0 (**b**) with ${e}_{m}=0.8$ (see Table 1 for parameter values). Each section represents different $TE$ with different colours.

By construction, $$0 \le TE \le f$$; thus, $$TE = 0$$ when either $$z = 0$$ or $$f = 0$$ and $$TE \to 1$$ when $$f \to 1$$ (i.e., almost all treated patients leave the hospital alive). $$TE = f$$ when $$z = 1$$, indicating that the treatment efficacy is not maximal even when the treatment has a curing efficiency of 100% but over a treatment duration of order of the patient lifetime in the hospital. The contour plots of $$TE$$ in ($$z$$, $$f$$) space are displayed in Fig. 3. Each section represents the different treatment efficacies ($$TE)$$ with different colours. High $$TE$$, particularly $$TE > 0.9$$ (small red area), requires a high value of $$f$$—i.e., a high fraction of treated patients leave the hospital alive (see Fig. 3). Different values of $$\nu$$ in Fig. 3 illustrate the effect of using different antibiotics.

### Transmission-controllable areas

Precaution compliance and antibiotic treatment are basic interventions to prevent disease transmission in hospitals. However, controlling the spread of CRKP remains crucial because of many important factors associated with CRKP infection and the limited understanding of the mechanisms underlying the transmission. To construct effective control measures, we must profoundly understand how fast the disease initially spreads and what components contribute to the transmission before implementing the interventions. In this study, two parameters were introduced to describe the epidemiological characteristics of disease transmission. First, the basic reproduction number in the absence of interventions ($R_{00}$) was used to delineate how the disease originally spreads and can be written in terms of the basic reproduction number for asymptomatic colonized patients ($R_{0c}$) and that for symptomatic colonized patients ($R_{0I}$).

17 $$\begin{array}{*{20}l} {R_{00} = \left[ {\frac{{mbb_{s} a^{2} }}{{\alpha_{min} }}} \right]\left[ {\frac{1 - x}{\gamma } + \frac{px}{\mu }} \right] = R_{0C} + R_{0I} } \\ \end{array}$$

The second is a parameter $\varepsilon$, defined by the ratio of $R_{0I}$ to $R_{00}$, that measures the relative contribution of symptomatic colonized patients in the transmission of the infection with respect to all colonized patients:

18 $$\begin{array}{*{20}l} {\varepsilon = \frac{{R_{0I} }}{{R_{00} }} = \frac{px\gamma }{{\left( {1 - x} \right)\mu + px\gamma }}.} \\ \end{array}$$

Furthermore, $\varepsilon$ can be adjusted by tuning the precaution contact effectiveness ($p$) so that lowering $p$ results in reducing the impact of symptomatic colonized patients in the transmission of infection in the hospital. This aspect must be considered together with the treatments when designing disease control strategies. Consequently, $R_{0}$ can be rewritten as a function of $R_{00}$ and $\varepsilon$ as follows:

19 $$\begin{array}{*{20}l} {R_{0} = \left[ {\frac{{\alpha_{min} \left( {1 - \delta } \right)^{2} }}{{\left( {1 - \delta } \right)\alpha_{min} + \delta \alpha_{max} }}} \right]\left[ {1 - \varepsilon + \varepsilon \left( {1 - {\varvec{TE}}} \right)} \right]R_{00} } \\ \end{array}$$

To determine sets of parameters for the combination of interventions, we constructed transmission-controllable areas of parameters based on $R_{0}$—that is, because the spread of infection is controlled for $R_{0} < 1$, the transmission-controllable area defines an ensemble of parameters such that $R_{0} < 1$. Therefore, to determine the boundary separating regions of sets of parameters that correspond to controllable and noncontrollable areas, we set $R_{0} = 1$ and solve the resulting equation for $\delta$ to obtain:

20 $$\begin{array}{*{20}l} {\delta = \frac{{\left( {2r - 1} \right)\alpha_{min} + \alpha_{max} - \sqrt {\left[ {\left( {2r - 1} \right)\alpha_{min} + \alpha_{max} } \right]^{2} - 4r\left( {r - 1} \right)\alpha_{min}^{2} } }}{{2r\alpha_{min} }},} \\ \end{array}$$

where $$r = \left[ {1 - \varepsilon + \varepsilon \left( {1 - TE} \right)} \right]R_{00}$$.

**Figure 4:**
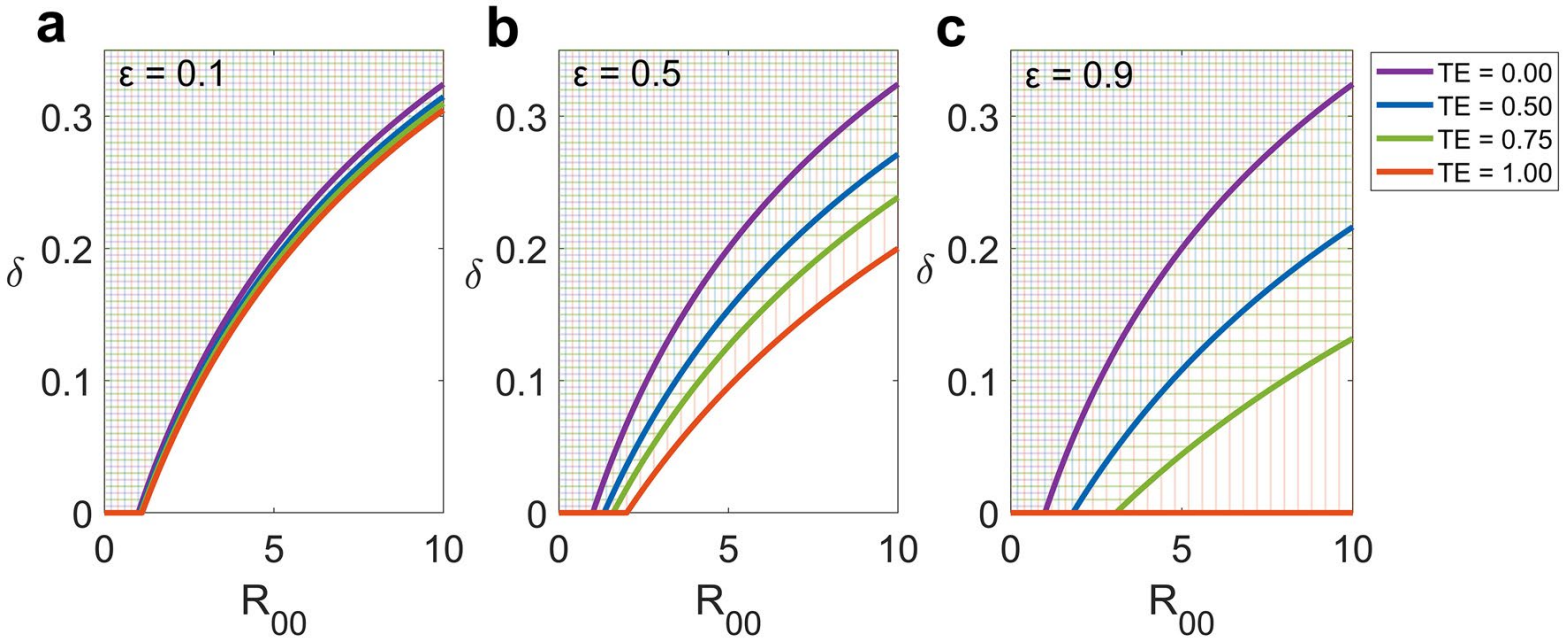
Transmission-controllable area. The transmission-controllable area (hatched areas above the lines) illustrates sets of parameters corresponding to the control of transmission with ${R}_{0}<1$. The solid line represents the ${R}_{0}$ = $1$ line separating the controlled (above) from the uncontrolled (below) areas. Different treatment efficacies ($TE$) are shown with different colours. Transmissions with $\varepsilon =0.1$, 0.5, and 0.9 are depicted in ($\mathbf{a}$), (**b**), and (**c**), respectively.

Four parameters—$R_{00}$,$\varepsilon$, the precaution compliance ($\delta )$, and the treatment efficacy ($TE$)—are used to determine the transmission-controllable area (Fig. 4). The $R_{0} = 1$ lines are calculated to separate sets of such parameters corresponding to $R_{0} < 1$ (transmission-controllable area) from $R_{0} > 1$ (uncontrollable area). In Fig. 4, the transmission-controllable areas are illustrated by the hatched areas above the $R_{0} = 1$ lines, and different treatment efficacies ($TE$) are represented by different colours.

Generally, when the infection spreads with $R_{00} > 1$, the minimum precaution compliance ($\delta$) must be increased to keep the spread controllable (Fig. 4). Additionally, when antibiotic treatment is implemented, the disease spread is more effortlessly controlled because enhancing the treatment efficacy enlarges the sizes of transmission-controllable areas. Interestingly, for transmission with $\varepsilon = 0.1$ (Fig. 4a), the sizes of transmission-controllable areas are almost the same from $TE = 0$ to 1, indicating that antibiotic treatment has no significant impact on reducing transmission with low $\varepsilon$. By contrast, the transmission-controllable areas are broader for transmission with a higher $\varepsilon$, particularly $\varepsilon = 0.9$ (Fig. 4c). This finding indicates that when transmission is dominantly driven by symptomatic colonized patients or a high $\varepsilon$, antibiotic treatment with a slightly higher $TE$ can considerably control the spread of disease. Therefore, the epidemiological characteristics of the transmission are unavoidable factors for designing intervention strategies. Within this framework, the transmission-controllable area provides potential control measures to combat the spread of and manage patients infected with CRKP in the hospital.

### Probability distribution of relapses

**Figure 5:**
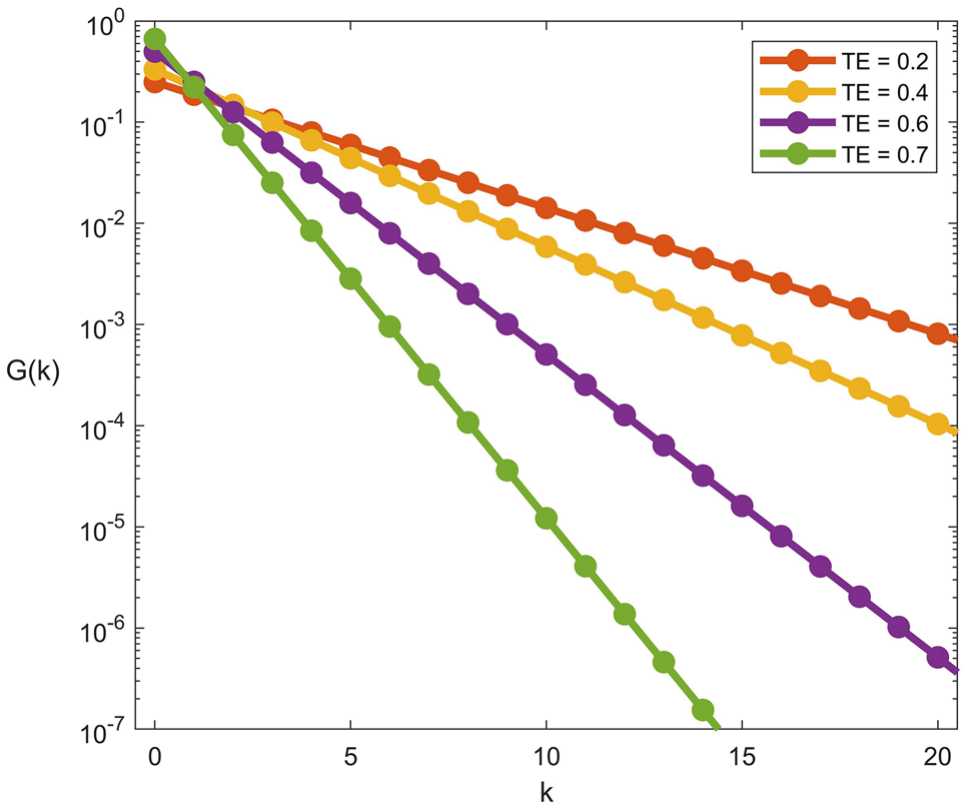
Probability distribution of relapses. *k* represents the number of times that a patient has experienced treatment failures or relapses. Different treatment efficacies ($TE$) are shown by different colours.

Treatment failure and rehospitalization due to a relapse of infection are significant factors contributing to continuing disease transmission in the hospital. In this section, the probability distribution, $G_{k}$, that symptomatic colonized patients have experienced $k$ treatment failures or relapses is simulated. For different treatment efficacies, $G_{k}$ *versus* $k$ follows a geometric distribution (see Eq. (31)) with the probability of relapse, $g = \left( {f - TE} \right)/\left( {1 - TE} \right)$, decreasing with treatment efficacy (Fig. 5).

### Impact of successive interventions

**Figure 6:**
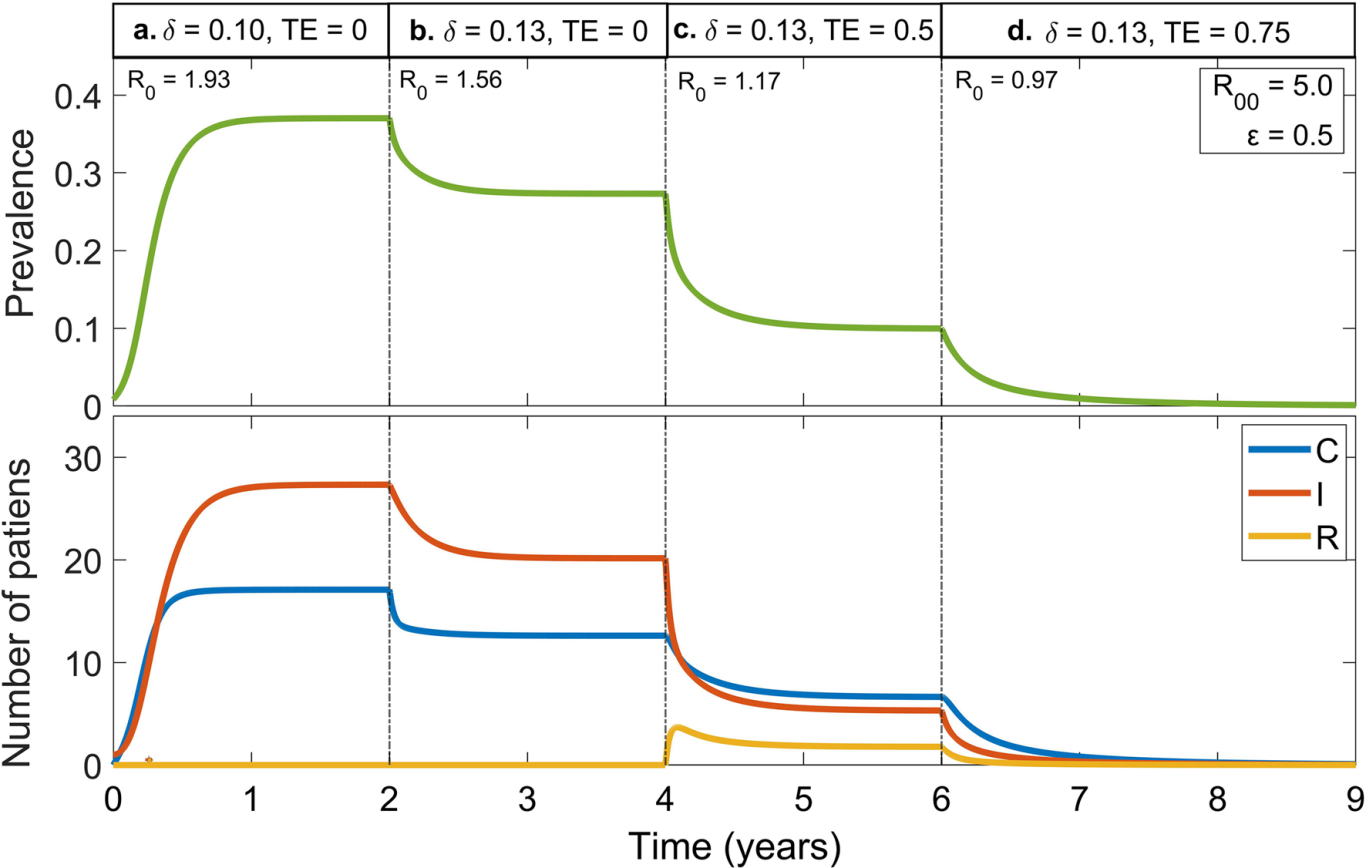
Impact of successive interventions on the time course of infection progression. The epidemic with no intervention (Panel **a**) is controlled by increasing precaution compliance (Panel **b**), followed by using the treatment on symptomatic colonized patients (Panel **c**), and finally by enhancing the treatment efficacy (Panel **d**). The impact of successive interventions on the epidemic time course is represented by the prevalence (above) and number of patients (below).

Finally, we simulated a scenario of successive interventions according to the time course of infection progression (Fig. 6). For illustrative purposes, the simulation starts with a single symptomatic colonized patient in the hospital, while all other patients are not colonized and susceptible to the infection, and no colonized patients from outside of the hospital are admitted. In the absence treatment, the infection is assumed to spread with $R_{0} = 1.93$ (Fig. 6, Panel a), and the total prevalence and numbers of asymptomatic and symptomatic colonized patients continuously increase over time up to the plateau steady state. The precaution compliance ($\delta$) is then increased from $0.1$ to $0.1$ 3 to mitigate the transmission (Panel b) by lowering $R_{0}$ to $1.56$. Subsequently, antibiotic treatment with a treatment efficacy ($TE$) of $0.5$ is set on. This drastically diminishes the prevalence and numbers of asymptomatic and symptomatic colonized patients in the hospital (Panel c). Because $TE$ is not sufficiently high, the number of relapsed patients in the hospital increases. As a final intervention, $TE$ is upgraded to $0.75$ at which point all the parameters are under the transmission-controllable area corresponding to $R_{0} < 1$. Thus, the infection in the hospital was completely eradicated (Panel d). Values of all parameters used in the simulation are summarized in Table 1.

## Discussion and conclusions

Carbapenem-resistant *Klebsiella pneumoniae* (CRKP) is a common pathogen associated with hospital-acquired infections. Controlling the CRKP spread is highly demanding because of most risk factors being associated with infections and a complicated mechanism hidden in the transmission. In the present study, we proposed a comprehensive transmission dynamics model for CRKP. Unlike previous studies^49,50^, our model included various compartments based on clinical characteristics—namely, uncolonized, asymptomatic colonized, symptomatic colonized and relapsed patients. The proposed model was then employed to improve understanding of how an incoming prevalence and antibiotic treatment shape transmission in a hospital. Additionally, to our knowledge, this study is the first to provide a transmission-controllable area allowing improved decision support for disease prevention and control.

The impacts of incoming prevalence on CRKP transmission in the hospital were first investigated. The total prevalence can be described by the prevalence generated by endogenous and exogenous colonized patients. Clearly, the within-hospital prevalence is zero when the basic reproduction number (${R}_{0}$) is less than one, while it progressively increases for ${R}_{0}>1$. Additionally, when more colonized patients are admitted, the prevalence increases regardless ${R}_{0}$. Likewise, the proportion of symptoms among colonized patients is independent of ${R}_{0}$ but depends on the incoming prevalence and fraction of symptomatic colonized patients admitted to the hospital. Additionally, using the weight parameters, we can identify which contributors, within-hospital or incoming prevalence, significantly affect the prevalence. Therefore, this information can help determine specific strategies to control the transmission. For example, we should either screen patients at admission, use contact precautions to reduce the spread of pathogens within the hospital or use a combination of both.

Our findings also indicate that the admission of patients directly influences transmission in the hospital^53,86^. Although ${R}_{0}$ can be reduced below unity using contact precautions, precaution compliance, or other interventions, the disease always persists in the hospital when colonized patients are constantly admitted to the hospital. Additionally, when ${R}_{0}$ is greater than one, the disease inevitably spreads in the hospital regardless of the admission of colonized patients, agreeing with previous modelling studies of the transmission dynamics of *Acinetobacter baumannii*^44^ and vancomycin-resistant *Enterococcus*^67^. Furthermore, this information may help support decision-making to implement active surveillance for CRKP carriers on admission, leading to early identification and isolation of colonized patients in control measures for disease prevention^73,87–89^.

Additionally, we introduced the treatment efficacy representing the effect of treatment applied on symptomatic colonized patients, including both the treatment duration and probability of successful treatment. Our results show that, although the probability of treating patients successfully is high, the disease can persist in the hospital if the treatment duration is too long compared with the time scale of transmission. The reason is that the symptomatic colonized patients treated with prolonged duration will stay longer in the hospital, leading to a high probability of transmitting pathogens to uncolonized patients^67,90^. However, they can still serve as a main source of CRKP in a hospital setting if they are treated with a short treatment duration but without effectiveness^38^. Therefore, patients with CRKP infection must be treated as swiftly and efficaciously as possible^47^. Attaining high treatment efficacy requires both a high probability of successful treatment and a high probability of leaving the hospital alive—i.e., a short treatment duration in the hospital (Fig. 3). This limitation can be overcome by changing to antibiotics that can more efficiently reduce the probability of patients becoming relapsed^32,91,92^.

Preventing the transmission of and managing infections associated with CRKP are challenging^93–95^. In our study, the transmission-controllable area was first proposed to provide criteria to design potential control measures. The disease will be eliminated in the hospital when values of parameters related to both intervention and transmission fall into the transmission-controllable area. We demonstrated that transmission with distinct epidemiological characteristics required different interventions. For example, the transmission dominantly driven by symptomatic colonized patients should be controlled with antibiotic treatment. The reason is that the impact of treatment increases when the disease is more transmissible, partly caused by the low effectiveness of contact precautions between staff and symptomatic colonized patients^86^. Additionally, the effectiveness of the intervention can be enhanced by improving precaution compliance. Although the minimum compliance rate must be increased if the disease originally spreads faster, this threshold can be reduced by increasing the treatment efficacy. In our simulation, we found that treatment with higher efficacy can significantly enlarge the transmission-controllable area (Fig. 4), resulting in a larger number of possible combinations of interventions that can control the transmission.

By contrast, to combat transmission in which symptomatic colonized patients are not the main drivers, precaution compliance is more critical than antibiotic treatment. The reason is that the impact of antibiotic treatment is less effective on such transmission, and even treatment with high efficacy cannot noticeably increase the size of the transmission-controllable area. Many studies have repeatedly demonstrated that precaution compliance is a crucial control strategy that substantially affects the endemic prevalence of nosocomial infections^49,50,67,68,96,97^. For example, endemic transmission could not be contained only by compliance with hand hygiene, ranging between 10 and 20% but could be eradicated when compliance was ameliorated to approximately 50%^98^. In practice, we must initially measure how disease originally spread in the hospital using the prevalence of colonized patients. Next, we tune the values of precaution compliance and/or treatment efficacy to make them fall into disease-controllable areas where the disease can be controlled. Additionally, we can use contact precautions to control disease spread. The reason is that adjusting the contact precaution will affect the main drivers who dominantly contribute to the transmission and change the size of the controllable area. Therefore, the transmission-controllable area is beneficial to design intervention strategies in which different combinations of antibiotic treatment and precaution compliance are effective for certain specific transmissions.

Treatment of infections associated with CRKP is very complicated, leading to various possible poor outcomes—e.g., relapse, persistence, or deterioration of symptoms^33,99^. Therefore, repeated retreatment of patients due to these unpleasant outcomes is inevitable. In this model, we can generate the probability distribution of symptomatic colonized patients with the history of the number of times a patient has experienced treatment failures or relapses (Fig. 5). We found that the distribution is directly governed by the treatment efficacy. Antibiotic treatment with higher efficacy lowers the probability that a patient will be retreated, leading to a decrease in the prevalence of CRKP in the hospital. By contrast, patients will have a greater probability of retreatment when receiving inefficacy and an inappropriate duration of antibiotic treatment^39,100^. Unfortunately, the supporting empirical data remain inadequate to construct the frequency of retreated patients attributed to antibiotic therapy failure. Only a few studies have provided details of case reports of patients who were retreated over one time^76,101,102^. Another group of patients who require retreatment is relapsing patients. The percentages of patients who subsequently become relapsing may vary in a substantial range, 0–65%, depending on several factors, such as antibiotics and duration of therapy^24,30,35,37,100,103,104^. This high rate of relapse causes patients to have repeated infections, which can reach up to four episodes of infections^39,76^. However, similar to the retreatment associated with failure of antibiotic therapy, the information containing the number of rehospitalized patients due to relapse is not sufficient for model validation. Fortunately, a few studies have investigated the recurrence of CRKP infections^38,103^. The number of episodes due to recurrent infections was counted using retrospective observational data. Interestingly, we found that the frequency of infection episodes was consistent with exponential behaviour, as described in our model. Note that recurrence was characterized as reinfection or relapse. Reinfection was defined as in patients for whom the recurrent isolates differed from the original genotype, whereas relapse indicates recurrence of infection with the same genotype.

In the present study, the proposed comprehensive model can describe CRKP transmission and assess the impact of disease control strategies on the transmission dynamics. However, the findings of this study are subject to several limitations. First, the model does not consider transmission through the hospital environment, which may act as a disease reservoir^105^. However, some empirical data demonstrated that hospital environment contamination marginally affects the spread of gram-negative bacteria^106^. Patient-to-patient transmission was also neglected in the model. Although this transmission route may occur when patients stay in the same unit, it is rare compared with staff-to-patient transmission^107^. Second, all the patients admitted to the hospital were assumed to have no history of the use of antibiotics. Disruption of the normal human gastrointestinal microbiota ecosystem due to antibiotic exposure predisposes patients to CRKP infections or colonization^108,109^. Therefore, this assumption might affect the transmission dynamics in the hospital setting. Third, although the development of resistant KP due to antibiotic exposure is interesting^110–115^, the modelling picture of the resistance of KP is not that clear and slightly complicated. For example, colonized asymptomatic patients carrying antibiotic-sensitive KP can additionally acquire antibiotic-resistant KP in their body after contacting staff contaminated with antibiotic-resistant KP^72,116^. Additionally, the competition between them in the same host remains vague. Patients with antibiotic-resistant KP infection who receive antibiotic treatment can become relapsed patients but with antibiotic-resistant KP because surviving bacteria develop resistance after antibiotic use^24,39,40^. Finally, antibiotics used for all treatments were assumed to be the same, as well as the treatment duration. In reality, the antibiotic treatment course should be adjusted according to the medical conditions of patients; this situation may also affect the prevalence of CPKP in the hospital^39,76^.

In conclusion, the understanding presented herein is valuable to describe the sophisticated mechanism of CRKP transmission and design more effective disease control programs. The influx of colonized patients, treatment efficacy of antibiotics, and characteristics of transmission are integral parts of disease control. The application of the proposed disease-controllable area is a novel strategy that may help us attain the maximum prevention and containment for CRKP transmission in the hospital setting.

## Methods

### Derivation of the basic reproduction number

To calculate the basic reproduction number using stability analysis, we consider only infective classes in the system of Eqs. (3)–(7) as follows:

21 $$\left\{ {\begin{array}{*{20}l} {\frac{dC}{{dt}} = y\Lambda + \frac{{\left( {1 - x} \right)ab\left( {1 - \delta } \right)S}}{{N_{s} }}C_{s} - \gamma C,} \\ {\frac{dI}{{dt}} = u\Lambda + \frac{{xab\left( {1 - \delta } \right)S}}{{N_{s} }}C_{s} - \left( {z\varphi + \mu } \right)I + rR, } \\ {\frac{dR}{{dt}} = ez\varphi I - rR, } \\ {\frac{{dC_{S} }}{dt} = \frac{{amb_{s} \left( {1 - \delta } \right)S_{s} }}{N}\left( {C + pI} \right) - \left[ {\left( {1 - \delta } \right)\alpha_{min} + \delta \alpha_{max} } \right]C_{s} .} \\ \end{array} } \right.$$

Note that $I = \mathop \sum \nolimits_{k = 0}^{{}} I_{k}$ and $R= \mathop \sum \nolimits_{k = 1}^{{}} R_{k}$. Next, we calculate the Jacobian, $J_{0}$, of Eq. (21) at the infection-free point, $I = C = C_{s} = 0$, $S = N$ and $S_{s} = N_{s}$. The determinant of $J_{0}$ is given by the following:

22 $$\begin{array}{*{20}l} {det\left( {J_{0} } \right) = \left| {\begin{array}{*{20}l} { - \gamma } & 0 & {\begin{array}{*{20}l} {0} &\quad {\frac{{\left( {1 - x} \right)ab\left( {1 - \delta } \right)N}}{{N_{s} }}} \\ \end{array} } \\ 0 & { - \left( {z\varphi + \mu } \right)} & {\begin{array}{*{20}l} {r } &\quad {\frac{{xab\left( {1 - \delta } \right)N}}{{N_{s} }}} \\ \end{array} } \\ {\begin{array}{*{20}l} 0 \\ {\frac{{amb_{s} \left( {1 - \delta } \right)N_{s} }}{N}} \\ \end{array} } & {\begin{array}{*{20}l} {ez\varphi } \\ {\frac{{ampb_{s} \left( {1 - \delta } \right)N_{s} }}{N}} \\ \end{array} } & {\begin{array}{*{20}l} {\begin{array}{*{20}l} { - r }\quad & 0 \\ \end{array} } \\ {\begin{array}{*{20}l} 0 & \quad { - \left[ {\left( {1 - \delta } \right)\alpha_{min} + \delta \alpha_{max} } \right]} \\ \end{array} } \\ \end{array} } \\ \end{array} } \right| } \\ \end{array}$$

23 $$\begin{aligned} & = \left[ {\gamma \left( {z\varphi + \mu } \right)r - \gamma rez\varphi } \right]\left[ {\left( {1 - \delta } \right)\alpha_{min} + \delta \alpha_{max} } \right] \\ & \,\,\,\,\, - \left[ {mbb_{s} a^{2} \left( {1 - \delta } \right)^{2} px\gamma r + \left( {1 - x} \right)mbb_{s} a^{2} \left( {1 - \delta } \right)^{2} \left( {\left( {1 - e} \right)z\varphi + \mu } \right)r} \right] \\ \end{aligned}$$

24 $$\begin{aligned} & = \gamma r\left[ {\left( {1 - e} \right)z\varphi + \mu } \right]\left[ {\left( {1 - \delta } \right)\alpha_{min} + \delta \alpha_{max} } \right] \\ & \,\,\,\, - mbb_{s} a^{2} \left( {1 - \delta } \right)^{2} \left[ {px\gamma r + \left( {1 - x} \right)\left( {\left( {1 - e} \right)z\varphi + \mu } \right)r} \right] \\ \end{aligned}$$

For the infection-free point to be stable it would be necessary that the (real parts) eigenvalues of $J_{0}$ are all negative, i.e., that $det\left( {J_{0} } \right)$, resulting from the product of the four eigenvalues of $J_{0}$, be positive. Thus, the basic reproduction number $R_{0}$ is defined such that $det\left( {J_{0} } \right) > 0$ for $R_{0} < 1$, i.e.,

25 $$\begin{array}{*{20}l} { det\left( {J_{0} } \right) > 0 \Rightarrow \frac{{mbb_{s} a^{2} \left( {1 - \delta } \right)^{2} \left[ {px\gamma r + \left( {1 - x} \right)\left( {\left( {1 - e} \right)z\varphi + \mu } \right)r} \right]}}{{\gamma r\left[ {\left( {1 - e} \right)z\varphi + \mu } \right]\left[ {\left( {1 - \delta } \right)\alpha_{min} + \delta \alpha_{max} } \right]}} < 1} \\ \end{array}$$

Therefore, $R_{0}$ is obtained by identification with the last expression of Eq. (25) as follows,

26 $$\begin{array}{*{20}l} {R_{0} = \frac{{mbb_{s} a^{2} \left( {1 - \delta } \right)^{2} }}{{\left[ {\left( {1 - \delta } \right)\alpha_{min} + \delta \alpha_{max} } \right]}}\left[ {\frac{px}{{\left( {1 - e} \right)z\varphi + \mu }} + \frac{1 - x}{\gamma }} \right] } \\ \end{array} .$$

### Steady state of the model

For ${R}_{0}>1$, the set of Eqs. (3)–(7) admits steady states for each class of patients and can be derived after calculations as

27 $$\left\{ {\begin{array}{*{20}l} {S^{*} = s_{0} + s_{1} I^{*} } \\ {C^{*} = c_{0} + c_{1} I^{*} } \\ {I^{*} = \left[ { - i_{1} + \sqrt {i_{1}^{2} + 4i_{0} i_{2} } } \right]/2i_{2} ,} \\ {R^{*} = \frac{{ze\varphi I^{*} }}{r} } \\ \end{array} } \right.$$

where,

28 $$\left\{ {\begin{array}{*{20}l} {s_{0} = \left[ {\left( {1 - u - y} \right) + \frac{1}{x}u} \right]N, } \\ {s_{1} = \frac{1}{\gamma }\left[ {\left( {1 - e} \right)z\varphi + \mu } \right]\left[ {\left( {1 - u - y} \right) - \frac{1}{x}\left( {1 - u} \right)} \right] - \left[ {\left( {1 - u - y} \right) + \frac{1}{x}u} \right], } \\ {c_{0} = \left[ { y - \frac{{\left( {1 - x} \right)}}{x}u} \right]N, } \\ \begin{gathered} c_{1} = \frac{1}{\gamma }\left[ {\frac{{\left( {1 - x} \right)}}{x}\left( {1 - u} \right) + y} \right]\left[ {\left( {1 - e} \right)z\varphi + \mu } \right] + \left[ {\frac{{\left( {1 - x} \right)}}{x}u - y} \right], \hfill \\ i_{0} = \frac{u\gamma N}{x}\left[ {\frac{{amb_{s} \left( {1 - \delta } \right)}}{N}c_{0} + \left[ {\left( {1 - \delta } \right)\alpha_{min} + \delta \alpha_{max} } \right]} \right] + \frac{{mbb_{s} a^{2} \left( {1 - \delta } \right)^{2} }}{N}c_{0} s_{0} , \hfill \\ i_{1} = \frac{1}{x}\left[ {\left( {1 - u} \right)\left( {\left( {1 - e} \right)z\varphi + \mu } \right) + u\gamma } \right]\left[ {\frac{{amb_{s} \left( {1 - \delta } \right)}}{N}c_{0} + \left[ {\left( {1 - \delta } \right)\alpha_{min} + \delta \alpha_{max} } \right]} \right], \hfill \\ \quad \quad - \frac{{amb_{s} \left( {1 - \delta } \right)}}{N}\left( {c_{1} + p} \right) \frac{u\gamma N}{x} - \frac{{mbb_{s} a^{2} \left( {1 - \delta } \right)^{2} }}{N}\left( {c_{0} s_{1} + s_{0} \left( {c_{1} + p} \right)} \right), \hfill \\ i_{2} = \frac{{amb_{s} \left( {1 - \delta } \right)}}{N} \frac{1}{x}\left( {c_{1} + p} \right)\left[ {\left( {1 - u} \right)\left( {\left( {1 - e} \right)z\varphi + \mu } \right) + u\gamma } \right] - \frac{{mbb_{s} a^{2} \left( {1 - \delta } \right)^{2} }}{N}s_{1} \left( {c_{1} + p} \right). \hfill \\ \end{gathered} \\ \end{array} } \right.$$

Additionally, symptomatic colonized patients ($I$) and relapsed patients ($R$) are classified into subpopulations corresponding to the number of times, *k,* that a patient has experienced treatment failure or relapses. Therefore, the steady states $I_{k}^{*}$ and $R_{k}^{*}$ (such that $I^{*} = \mathop \sum \nolimits_{k = 0}^{{}} I_{k}^{*}$ and $R^{*} = \mathop \sum \nolimits_{k = 0}^{{}} R_{k}^{*}$) are computed as follows:

29 $$\begin{array}{*{20}l} {\left\{ {\begin{array}{*{20}l} {I_{k} = \left[ {\frac{f - TE}{{1 - TE}}} \right]^{k} I_{0} } \\ {R_{k} = \frac{ze\varphi }{r}I_{k - 1} } \\ \end{array} } \right.} \\ \end{array}$$

Finally, using the relation $I^{*} = \mathop \sum \nolimits_{k = 0}^{\infty } I_{k}^{*}$, we obtain,

30 $$\begin{array}{*{20}l} {I_{0} = \left( {\frac{1 - f}{{1 - TE}}} \right)I^{*} } \\ \end{array}$$

Now, plugging back Eq. (30) into Eq. (29), we obtain,

31 $$\begin{array}{*{20}l} {I_{k} = I^{*} G_{k} \Rightarrow G_{k} = \left( {1 - g} \right)g^{k} ; g = \left( {\frac{f - TE}{{1 - TE}}} \right)} \\ \end{array}$$

where $G_{k}$ is the normalized (i.e., $\mathop \sum \nolimits_{k = 0}^{\infty } G_{k} = 1$) probability distribution that a symptomatic colonized patient has experienced *k* relapses, and $g$ is the probability of relapse.

### Simulation details

In this study, patient-to-patient and staff-to-staff transmissions were not considered, and the patient and staff populations were assumed to be homogeneous. Additionally, the number of patients in the hospital was kept constant such that the total number of admissions equals that of discharge from the hospital:

32 $$\begin{array}{*{20}l} {\Lambda + rR = \gamma S + \gamma C + \left( {z\varphi + \mu } \right)I.} \\ \end{array}$$

Additionally, we assume that the dynamics of contamination in staff are fast compared with those in the patient population. Therefore, we consider the dynamics of contamination in staff at the steady state with the number of contaminated staff members given by the following:

33 $$\begin{array}{*{20}l} {C_{S} = \frac{{\lambda_{S} N_{S} }}{{\lambda_{S} + \left[ {\left( {1 - \delta } \right)\alpha_{min} + \delta \alpha_{max} } \right]}},} \\ \end{array}$$

to be used in Eq. (1).

Additionally, determining the death rate ($\mu$) is slightly challenging because the reported deaths include several cases in which the patients have severe underlying disease or comorbidity. Hence, the attributable mortality rate was used to reduce this ambiguity^5,117^. The attributable mortality rate provides the percentage of infection-related deaths during a length of hospital stay. Therefore, $\mu$ was calculated as follows:

34 $$\begin{array}{*{20}l} {\mu = \frac{{ - {\text{ln}}\left( {1 - 0.01 \times {\text{attributable mortality }}\left( {\text{\% }} \right)} \right)}}{1/\varphi }.} \\ \end{array}$$

In this study, the length of hospital stay for symptomatic colonized patients was assumed to be equal to the treatment duration ($1/\varphi$).

For simulations, the transmission dynamics of CRKP were calculated by solving the set of ODE Eqs. (3)–(9) using the Euler method. Initially, a symptomatic colonized patient was introduced to the hospital. He or she transmitted the pathogens via staff, and the contaminated staff then spread the disease to other patients. The time step used is 0.001 days. The descriptions and values of all parameters used in the model are summarized in Table 1. Note that the values used in simulations were selected from the ranges extracted from various studies. Furthermore, to investigate the effects of factors on the transmission dynamic, particularly at equilibrium, we used steady-state equations by solving ODEs numerically. In the case of no treatment, $\varphi$ and $z$ are set to zero; in the case of no incoming prevalence, $u$ and $y$ are set to zero. A change in $R_{0}$ resulted from varying $\delta$. All the figures and calculations were generated using MATLAB software (version R2020b; The MathWorks, Inc).
